# A voluntary conservation agreement reduces the risks of lethal collisions between ships and whales in the St. Lawrence Estuary (Québec, Canada): From co-construction to monitoring compliance and assessing effectiveness

**DOI:** 10.1371/journal.pone.0202560

**Published:** 2018-09-21

**Authors:** Clément Chion, Samuel Turgeon, Guy Cantin, Robert Michaud, Nadia Ménard, Véronique Lesage, Lael Parrott, Pierre Beaufils, Yves Clermont, Caroline Gravel

**Affiliations:** 1 Department of Natural Sciences, Université du Québec en Outaouais, Gatineau, Québec, Canada; 2 Saguenay–St. Lawrence Marine Park, Parks Canada, Tadoussac, Québec, Canada; 3 Maurice-Lamontagne Institute, Fisheries and Oceans Canada, Mont-Joli, Québec, Canada; 4 Group for Research and Education on Marine Mammals (GREMM), Québec, Québec, Canada; 5 Departments of Earth, Environmental and Geographic Sciences and Biology, University of British Columbia, Kelowna, British Columbia, Canada; 6 Marine Safety & Security, Transport Canada, Ottawa, Ontario, Canada; University of California Santa Cruz, UNITED STATES

## Abstract

Lethal collisions with ships are limiting the recovery of several at-risk whale species worldwide. In the St. Lawrence Estuary (Quebec, Canada), the *endangered* blue whale and *of special concern* fin whale are among the migratory species subject to collisions with large ships. In 2011, a working group composed of representatives from the maritime industry, the government, non-governmental organizations, and academia was created to explore solutions to mitigate ship-whale collisions in the St. Lawrence Estuary. Adopting an adaptive risk management framework, the working group took advantage of the best available scientific data and tools to co-construct realistic collision mitigation options and evaluate their likely benefits for whale conservation and costs for the industry. In 2013, the working group recommended the implementation of voluntary measures to mitigate collision risks, consisting of a slow-down area, a no-go area, and a caution area; a recommended route was added in 2014. Along with the voluntary framework, the working group agreed to continuously monitor compliance with and assess effectiveness of these mitigation measures. After the fourth year of implementation, voluntary measures showed encouraging results, with a reduction of up to 40% of lethal collision risks with fin whales in the highest density area. This reduction in risk is mainly related to ship speed reduction in the slow-down area from 14.1 ± 2.6 knots in 2012 to 11.3 ± 1.7 knots since 2014. The presence of a mandatory pilotage area overlapping with the slow-down area was instrumental to facilitate communication about the mitigation measures, with the pilotage corporation sitting as a regular member of the working group. This resulted in significantly slower speeds in the slow-down area for ships with a pilot from the pilotage corporation onboard compared to those without (-0.8 knots, p-value < 0.001). It is also likely to explain the weaker compliance of the maritime industry with the no-go area located outside of the mandatory pilotage area. Other factors of success include: the continuous dedication of the government to a voluntary and transparent participatory process; the use of available data, tools and institutions; the presence of an environmental certification program representative in the working group; and the adoption by consensus of an adaptive risk management approach. The traditional regulatory approach to conservation is often blamed for its focus on deterring negative behaviors, doing nothing to encourage and reward positive ones. In agreement with other case studies, the benefits of the voluntary measures implemented in the St. Lawrence Estuary include the pro-active commitment from the industry (which is likely to reduce conflicts with regulators), the greater flexibility and freedom that allowed to come up with cost-effective and tailored-made mitigation measures, and the fast achievement of conservation gains. More importantly perhaps, the human and working capital built throughout the concertation process have the potential to be a fundamental cornerstone in dealing with more complex issues such as the chronically increasing level of underwater noise in whale habitats.

## Introduction

The anthropogenic pressure on marine ecosystems has gained growing attention from conservation agencies and the general public over the last two decades. In particular, shipping risks including collisions with whales [1] and impacts of underwater noise [2] are social-ecological issues that have triggered mitigation endeavors all around the world [3–5]. Focusing on the acknowledged issue of collisions between merchant ships and large whales [6], the perspective of an increase in commercial shipping [7,8] is calling for effective mitigation measures to reduce collision risks, especially for endangered cetacean species [9,10].

Several technological mitigation options have been put forward to reduce collision risks and whale lethality [3,11], including passive acoustic monitoring systems [12] and infrared vision systems [13]. However, scientific evidence has led to the implementation of operational measures to mitigate collision risks all around the world, including the establishment of areas to be avoided [10], speed restrictions [14], and recommended routes (rerouting) [5]. It is now well documented that the probability of a collision being lethal to a whale increases with ship speed and length [1,14–16]. When it is safely possible to do so, the priority is currently given to routing measures as they appear to be more effective so far to mitigate collisions [5,6,11]. The development and planning of such operational measures have been proposed in several areas using data about whale movements and densities and shipping patterns [17].

The effectiveness of operational measures is a function of the degree of compliance with the measures, and adequacy of the area and time chosen for their implementation. A full compliance of the shipping industry with a given measure could be achieved, but if the measure is implemented in a low risk area or at a time when whales are less present, then it could be inefficient. Therefore, there is a need to assess and monitor both compliance and effectiveness to improve them and allow for adaptive management [18].

The traditional regulatory approach to conservation is often blamed for its focus on deterring negative behaviors, doing nothing to encourage and reward positive ones [19]. Voluntary measures have been increasingly used by regulators for several decades to enhance environmental protection. The potential benefits of voluntary measures include the pro-active commitment from the industry which is likely to reduce conflicts with regulators, the greater flexibility and freedom to come up with cost-effective and tailored-made solutions, and the fast achievement of environmental objectives [20,21]. Many researchers have been investigating the conditions of success of voluntary agreements to protect endangered species [19,20,22]. These endeavors indicate that successful conservation of endangered species using a voluntary framework is supported by such factors as the threat of regulation (“stick approach”), incentives (“carrot approach”), and the provision of assurances regarding future regulation [19].

Several institutional mechanisms have been used to come up with operational mitigation measures, whether top-down or bottom-up approaches using regulatory [23] and voluntary processes [5,24]. However, the compliance with voluntary operational measures and their effectiveness are rarely assessed in a consistent way (*e*.*g*. limited surveillance, unreliable data), with only a fraction of those cases that have been assessed (*i*.*e*. 20 out of a total of 114) being proven effective [18]. Moreover, although the potential of and need for voluntary approaches in marine resources conservation are increasingly acknowledged [18,25,26], it has been reported that the voluntary agreements implemented so far to mitigate collision risks are less effective than statutory ones, highlighting the importance of monitoring and enforcement to make such measures successful [23,27,28].

In this context, our first objective is to report on a case study that demonstrates the value of voluntary measures at reducing collision risks, using a comprehensive methodology to assess compliance and effectiveness. We also provide a perspective on key factors that play an important role in the successful implementation of a voluntary conservation approach. Finally, we identify ways to further improve the success of these measures.

## Case study

### Social-ecological context: Collision risks

**Fig 1:**
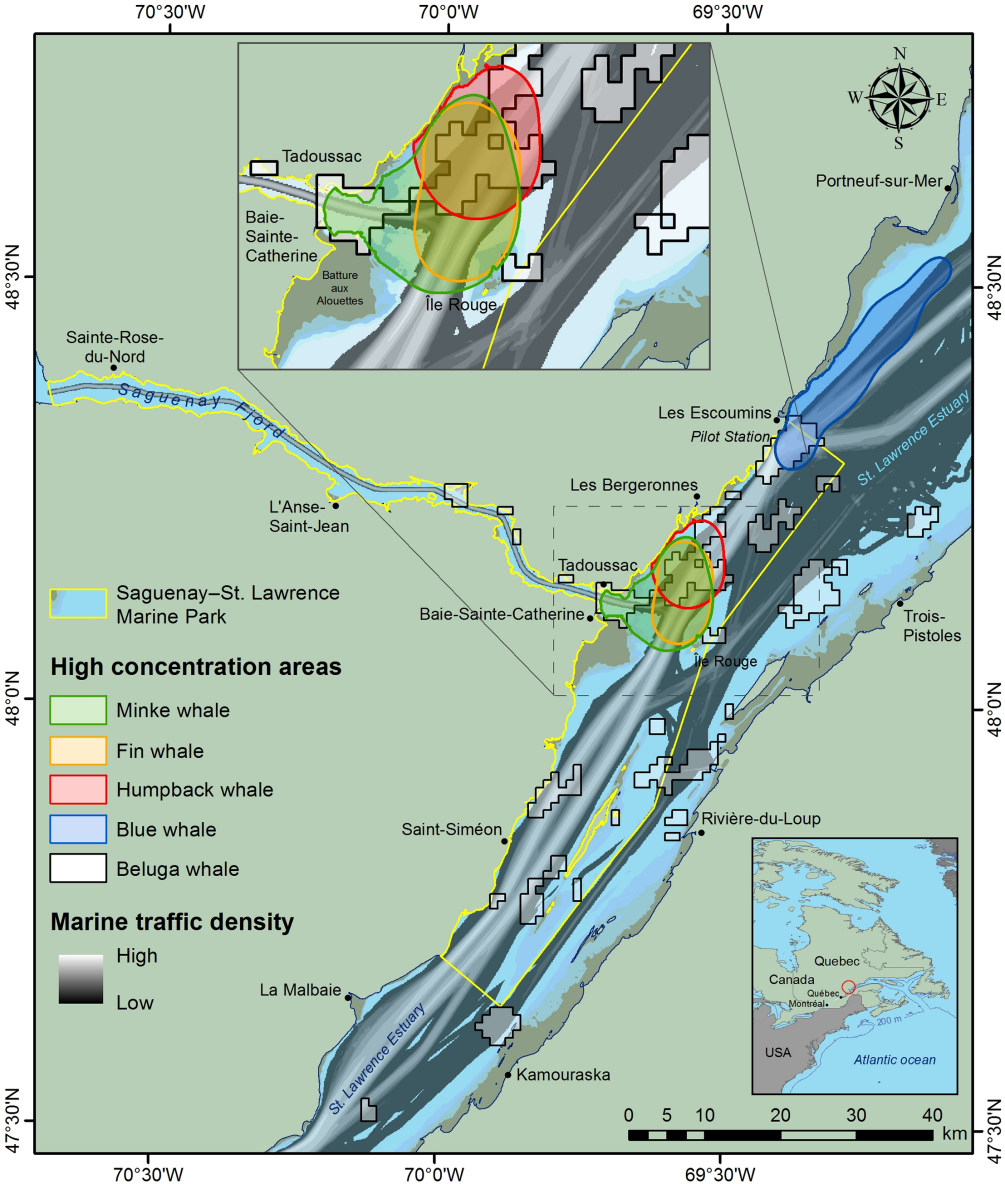
Study area. St. Lawrence Estuary and the Saguenay River, Québec, Canada (Figure created by the authors. Data sources: Canadian Hydrographic Service; Parks Canada; Fisheries and Oceans Canada; Group for Research and Education on Marine Mammals; and ESRI).

This study is conducted in the St. Lawrence Estuary (SLE), in and around the Saguenay–St. Lawrence Marine Park in Québec, Canada (Fig 1). This area is a feeding ground (mainly from May to October) to several cetacean species listed under the Canadian Species At Risk Act [29] including the migratory blue whale (*endangered*) and fin whale (*of special concern*) for which collisions are identified as a major threat limiting their recovery [30]. The resident population of the St. Lawrence Estuary beluga (*endangered*) also frequents this area which overlaps its critical habitat [31]. The SLE is also a relatively busy commercial waterway to the heart of North America. Approximately 5000 transits of merchant ships are recorded each year [32] in the SLE where a mandatory pilotage zone exists upstream of Les Escoumins (Fig 1). Accordingly, most ships in transit are required to get an expert pilot onboard to ensure safe navigation [33]; Mandatory pilotage is administered by the Laurentian Pilotage Authority and the service between Quebec City and Les Escoumins (Fig 1) is ensured by the Corporation of the Lower St. Lawrence pilots (CLSLP, also referred to as pilotage corporation thereafter). While commercial shipping occurs year-round, activity increases during the ice-free months. In addition to this, a well-established whale-watching industry operates from May to October in this region, and mainly focuses on baleen whales such as minke, fin, humpback, and blue whales [34]. While whale distributions and densities are heterogeneous in the region, they are generally higher between Les Escoumins and Tadoussac where merchant ships transit and the traffic lanes are located (Fig 1). This overlap between merchant ship traffic and whale distributions (along with whale-watching vessels) increases the risks of collision between large ships and whales (and with whale-watching vessels). In this context, conservation managers wanted to identify and implement appropriate measures to mitigate the risks of collisions between ship and whales in the SLE.

Following decades of scientific data collection and research on the ecology of the St. Lawrence whales and navigation activities in and around the Saguenay–St. Lawrence Marine Park [34], spatial databases for whale and ship distribution and density patterns along with decision support tools [35–37] were developed to inform a multi-stakeholder process aiming at mitigating collision risks [4].

### Collaborative approach

In order to explore realistic and effective mitigation measures to reduce collision risks between ships and baleen whales in the SLE without compromising safety and marine activities, a working group called The *Groupe de travail sur le transport maritime et la protection des mammifères marins* (Group for marine transportation and marine mammal protection, referred to as working group thereafter) was created in 2011 [4]. Based on the best practices in stakeholder participation [38], this working group was created out of the *Comité Concertation Navigation*, an existing concertation table dedicated to sustainable navigation in the St. Lawrence waters that originates from a Québec-Canada agreement. The working group was later designated as an independent subgroup of this concertation table to which it reports its activities and results on a yearly basis.

The working group is composed of a diversity of representatives from the federal government, shipping industry, pilots, academia, and economic and environmental non-governmental organizations (NGOs) (S1 Table). The composition of the working group is driven by the issues to be addressed, and can be modified on an *ad hoc* basis to include additional external expert observers, or stakeholders when deemed appropriate. Early in the process, the working group agreed to rely on the best scientific knowledge, data and decision-support tools available to inform decisions and recommendations in a context where detecting and recording collisions is very challenging [1,16,39]. Although strikes with whales have been reported in the area with various boat types (data partially published in [1]), the working group decided to base their working process on risk management since an exhaustive count of collision events could not be achieved. The working group also based their governance on consensus through concertation, from problem definition to problem solving [40].

**Fig 2:**
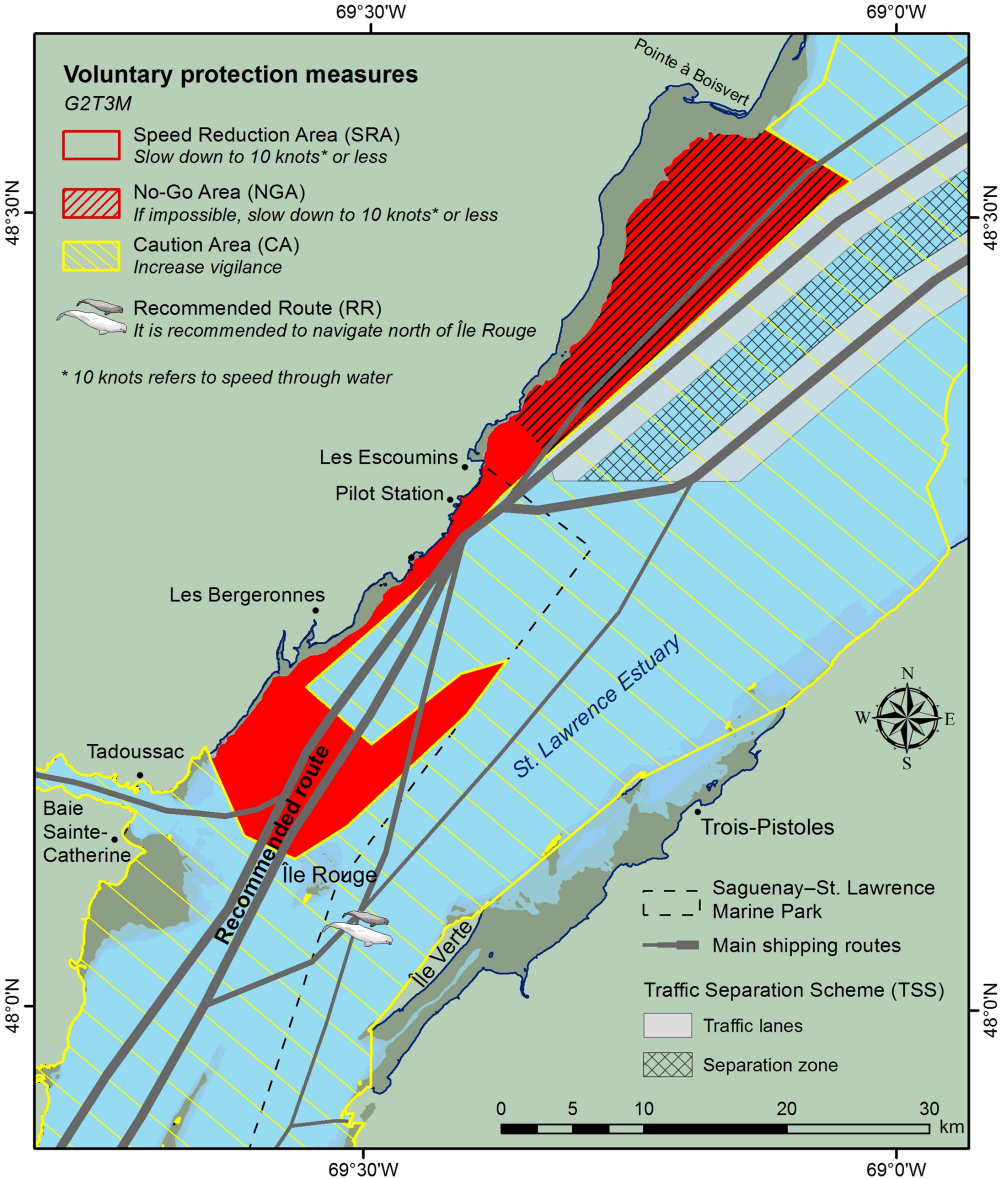
Voluntary operational measures to mitigate collisions between ships and whales in the St. Lawrence Estuary as of 2017. (Figure created by the authors. Data sources: Canadian Hydrographic Service; Parks Canada; Fisheries and Oceans Canada; and ESRI).

A series of meetings were conducted to understand the operational constraints of both the shipping industry and the pilots in terms of safety and time buffered into each transit that could be steered toward mitigation measures. At the same time, the best science available about change in collision risk with vessel type, length and speed, and avoidance behavior and capacity for whales were presented. Following these initial workshops, the Marine Mammal and Maritime Traffic Simulator (3MTSim) [35,37] was calibrated based on experts’ knowledge to explore 10 scenarios co-constructed by the working group members likely to reduce collision risks without compromising safety at sea and maritime activities [4]. All 10 scenarios were combinations of no-go areas, speed reduction areas, and recommended routes [41]. After two iterations of scenario co-construction and evaluation using 3MTSim and comprehensive analyses of scientific data on the ecology of marine mammals and marine traffic, the working group reached a consensus early 2013. This consensus was based on the expected conservation gains in the perspective of the economical and logistical constraints brought forward by the maritime industry and St. Lawrence pilots. To be consistent with the participatory nature of the process, the working group decided to recommend the implementation of a set of provisional and voluntary operational measures to mitigate collision risks in the region (Fig 2), based on the previous phases of scenario co-construction and evaluation [41].

### Components of the voluntary agreement

Two years after the working group inception and about six months after the recommendation to implement provisional voluntary operational measures to mitigate collision risks in the SLE, the first version of these measures was implemented in June 2013 (the latest version of the voluntary measures is presented in Fig 2 [42]).

From the first version on, in conformity with the International Maritime Organization’s guidelines [6], the set of operational mitigation measures have consisted of a no-go area (NGA), a speed reduction area (SRA) at 10 knots or below, and a caution area that apply to the shipping industry only. The 10-knot speed limit was selected as being the best trade-off to reduce the probability of a collision being lethal for a whale (~31%, [16]) while maintaining ship maneuverability [43]. The NGA was designed mostly to prevent the occurrence of merchant ships in a feeding area of the endangered blue whale [44]. Ships that are unable to transit outside the NGA (*e*.*g*. for safety reasons) are asked to maintain their speed below 10 knots (Fig 2). This is consistent with standards that promote the avoidance of whale high-density areas over speed reduction to mitigate collision risks [3,5,11,16]. The SRA corresponds to a feeding area recurrently used by other whale species, which is caused by particular bathymetric features and water mass circulation [45–47]. Because currents are locally strong in the SRA due to tides, it is hard for a mariner to maintain a constant speed over ground (SOG). Therefore, following the recommendations of the expert pilots, the working group decided to define a speed through water (STW) limit for ships in the SRA rather than SOG (S1 File).

The recommended route (RR) north of Île Rouge and along the north shore of the SLE (Fig 2) has been implemented in 2014 to keep traffic away from high-use areas for females and young belugas [48–50] and maintain the areas south of Île Rouge relatively quiet [51–53]. Between 2013 and 2017, following an adaptive management framework, the working group revised these measures and proposed minor changes and adjustments based on science [52], seamanship considerations, and consistency with other measures already implemented in the area.

## Materials and methods

### Data

Data from the Automatic Identification System (AIS) have become a *de facto* standard to monitor shipping activities and detect potential behavioral responses of ship captains following the implementation of conservation measures [27,54–56].

In the SLE, all ships affected by the voluntary measures are required to be fitted with an AIS [57], making it a suitable data source for monitoring purpose. AIS data in the marine park area is provided by Parks Canada through a network of three land-based antennas ensuring a spatial coverage that encompasses the area of the measures (Fig 2). The AIS data archiving station includes a real-time speed conversion module (SOG to STW) that is based on a surface current prediction model provided by Fisheries and Oceans Canada and ship course [58]. The spatial resolution of the model is 400 m and it is updated every 60 minutes, with data at a shorter time scale calculated by linear interpolation. There is a 0.7-knot uncertainty in the estimation of ship STW from ship SOG (drawn from AIS data) and the surface current model [58], the latter being responsible for the larger part of it due to modelling errors and downscaling effects (S1 File).

Despite their numerous advantages, AIS data suffer several known pitfalls that must be considered during processing and interpretation. For instance, limits to AIS coverage, corrupted data, and vessels not carrying AIS systems may prevent a comprehensive count of ship transits in a given area [56]. Therefore, a pre-processing exercise was carried out on the AIS data to remove any poor data points (*e*.*g*. incomplete transit, erroneous speed, bad position) for the study area and over the study period.

Another important characteristic of AIS data to account for is the transmission/reception rate. For a given AIS data transmission rate, slower ships will be over-represented in the dataset compared to faster ones, which may introduce a negative bias when assessing for instance the average speed of ship transits in a given area [23,55]. Consequently, adequate data pre-processing and metrics are needed to avoid introducing biases in the analyses [23,56].

To evaluate the conservation benefits from compliance of the shipping industry with voluntary measures, we used the same data on whale movement and distribution patterns as those used by the working group to design the operational mitigation measures (Fig 2). These data are fully presented in [37] (Table 1), and draw from a 25-year portrait of the summer spatial distributions, densities and movements of four baleen whale species (*i*.*e*., minke, blue, fin and humpback whales). These data include 547 baleen whales sightings from transect surveys, 140 focal follows tracked from land-based stations, 80 whales tracked by VHF, and more than 32 000 whale sightings from whale-watching boats [37]. The core area used by each of the target species are shown in Fig 1. While abundance and areas of concentration may vary seasonally or among years, the lack of quantitative analysis of this variability precluded it from being considered in the analyses.

**Table 1:** Results of a Generalized linear mixed model highlighting the statistically significant impact of the voluntary measures on ship distance-weighted average speed (DWAS) in the speed reduction area (SRA). The intercept contains transits DWAS when the measures are inactive (2012–2016).

	DWAS (I_5_)		
* *	*Estimate*	*Conf*. *int*.	*p-value*
**Fixed Parts**			
(Intercept)	14.1	14.0 – 14.1	< .001
SRA (active)	-2.8	-2.9 – -2.7	< .001

### Methods

#### Compliance assessment

In the context of voluntary conservation agreements, we argue that the evaluation of compliance of a community of users with a set of management measures must acknowledge all the individual efforts leading to environmental gains. This appears to be even more important in our context of risk management where the actual impacts of compliance on the whale species to protect at both the individual and population levels are subject to uncertainties. In this perspective, we adopted a portfolio of indicators to acknowledge and communicate the multiple dimensions of compliance. This allows for a better identification of follow-up actions to enhance conservation and keep the members of the community motivated to embrace a process of continuous improvement.

All compliance analyses were conducted using AIS data available for years 2012 to 2016. In the case of the SRA where ships are recommended to navigate at STW of 10 knots or less, we developed the following portfolio of indicators to capture the multiple dimensions of compliance:

1. Strict compliance (I_1_): To be considered strictly compliant, a transit must have all its AIS data in the SRA at 10 knots or less (STW). Indicator I_1_ is considered a severe indicator of compliance because if only one AIS point reported from a transit had a speed greater than 10 knots, the whole transit of 5.4 nautical miles was classified as non-compliant. Therefore, to further assess compliance, we also proposed I_6_ and I_7_ detailed below.
2. Slow-down effort (I_4_): Ship speed variation before entering the SRA (3 km buffer zone) *vs*. within the SRA. We used average speed in both areas to calculate the variation.
3. Unbiased average compliance (I_5_): Distance-weighted average speed (DWAS) of all AIS data of a transit in the SRA [55]. DWAS is used to compensate for the bias induced by the over-representation of AIS data at lower speeds and by variations in the AIS signal transmission/reception rates.
4. Level of non-compliance (I_6_, I_7_): Following the methodology proposed in [23], we calculated indicators I_6_ and I_7_ to assess compliance with the 10-knot speed limit in the SRA. Accordingly, we calculated the percentage of the total transit distance traveled within the SRA at speeds greater than 10 knots [23] called I_6_. However, we used the distance-weighted average instead of the basic average when calculating the speed of non-compliant segments of ship routes in the SRA (I_7_).

Additionally, to statistically analyze ship compliance in the SRA, we used Generalized linear mixed models (GLMMs) using RStudio version 1.1.442 with R version 3.4.4. GLMMs analyses were conducted with the function *lmer* of the *lme4* package [59]. Confidence intervals and p-values (via Wald-statistics approximation) were calculated with the function *sjt*.*lmer* of the *sjPlot* package [60]. We proceeded by running GLMMs (using *year* as the random-effect variable) to answer the three following questions:

1. *Do voluntary measures influence ship speed in the SRA*? We used a GLMM where the dependent variable was transits DWAS through water in the SRA, with one binary independent variable (voluntary measures are active *vs*. inactive) as fixed part. The intercept contains DWAS when the measures were inactive.
2. *Is there any inter-annual variability of ship speed when voluntary measures are active*? We used a GLMM where the dependent variable was transits DWAS through water in the SRA, using the variable year when measures were active as the independent variable. The intercept contains DWAS when the measures are inactive. Additionally, we used the non-parametric Kolmogorov-Smirnov test (*ks*.*test* function of the R *stats* package) to test for any statistically significant difference in compliance over the years. This analysis is meant to assess whether the maritime industry’s commitment to a continuous improvement in compliance over the years is achieved.
3. *Which factors explain ship speed variability when voluntary measures are active*? To determine if some factors statistically explain variability in the compliance pattern with the speed limit in the SRA, we used a GLMM framework with transits DWAS as the dependent variable and considered the following independent variables:

- *Ship class* (3 categories): cargo, passenger, or tanker (included in the GLMM intercept).
- *Ship’s country of registration* (2 categories): Canadian or international flag. Let’s note that all international ships have a pilot from the CLSLP onboard in the SRA.
- *Pilotage* (2 categories): Whether or not a pilot from the CLSLP was onboard during the transit through the SRA.
- *Transit direction* (2 categories): upstream (southward) or downstream (northward).

For the NGA or where ships are asked to maintain a speed of 10 knots or less, we assessed:

4 Non-compliance (I_2_): proportion of transits travelling (completely or partially) through the NGA.
5 Compliance: DWAS (I_5_) for transits using completely the NGA.

For the RR we assessed:

6 Strict compliance (I_3_): proportion of transits travelling north of Île Rouge.

#### Effectiveness and consequences of mitigation measures

Evaluating the effectiveness of mitigation measures is important for communication and outreach purposes and can serve as a source of motivation to promote and enhance compliance. Effectiveness was assessed by comparing the risks of lethal collisions before and after the implementation of the voluntary mitigation measures. We took August 2012 as the reference period for this analysis instead of the 2012–2016 period when the voluntary measures are inactive (*i*.*e*. from November to April) because whales are mostly present in the study area from May to October; August 2012 is the only period covered in our AIS dataset during the “whale season” when the measures are not active (before their implementation). We used a probabilistic approach originally developed to assess the risks of lethal collisions with North Atlantic Right Whale in the Bay of Fundy and on the Scotian Shelf [17].

This approach requires data on ship positions and speeds, and an estimation of the spatial distribution of target whale species. Whereas shipping data are available through AIS on a yearly basis in our study area, we rely on an historical 25-year dataset for whale distributions.

The 6-step spatial analysis procedure performed to assess the effectiveness of the voluntary operational measures is described in the supplementary material (S2 File). For a given species and area, it is possible to evaluate the percentage of reduction of the lethal collision risks (referred to as effectiveness) by comparing the risk maps before and after the implementation of the voluntary mitigation measures.

We also estimated the consequences of mitigation measures for the maritime industry by calculating ship transit times between Les Escoumins and Saint-Siméon (Fig 1) before and after mitigation measures were put in place. We chose these spatial limits to encompass the area of their normal route where ships are requested to slow down to take onboard an expert pilot from the CLSLP, and included an additional spatial buffer to account for eventual speed compensations to catch up for delays. The drop in the amount of AIS data captured downstream of Les Escoumins and upstream of Saint-Siméon, indicative of the spatial coverage of land-based AIS receivers, also guided the choice of spatial limits for this. Due to data availability (spatial and temporal coverage), we used August 2012 as a reference (only AIS data available during the summer “whale season” when the measures are inactive) and 2016 when the measures were active. Therefore, this preliminary analysis is presented to highlight the order of magnitude of the measures cost for the industry and should be regarded as a first effort in this direction.

## Results

In this section, we present the results of compliance with the three voluntary measures (Fig 2), namely the SRA, the NGA, and the RR along with their effects both on conservation and shipping activities.

### Speed reduction area (SRA)

#### Effect of the voluntary measures in the SRA

**Fig 3:**
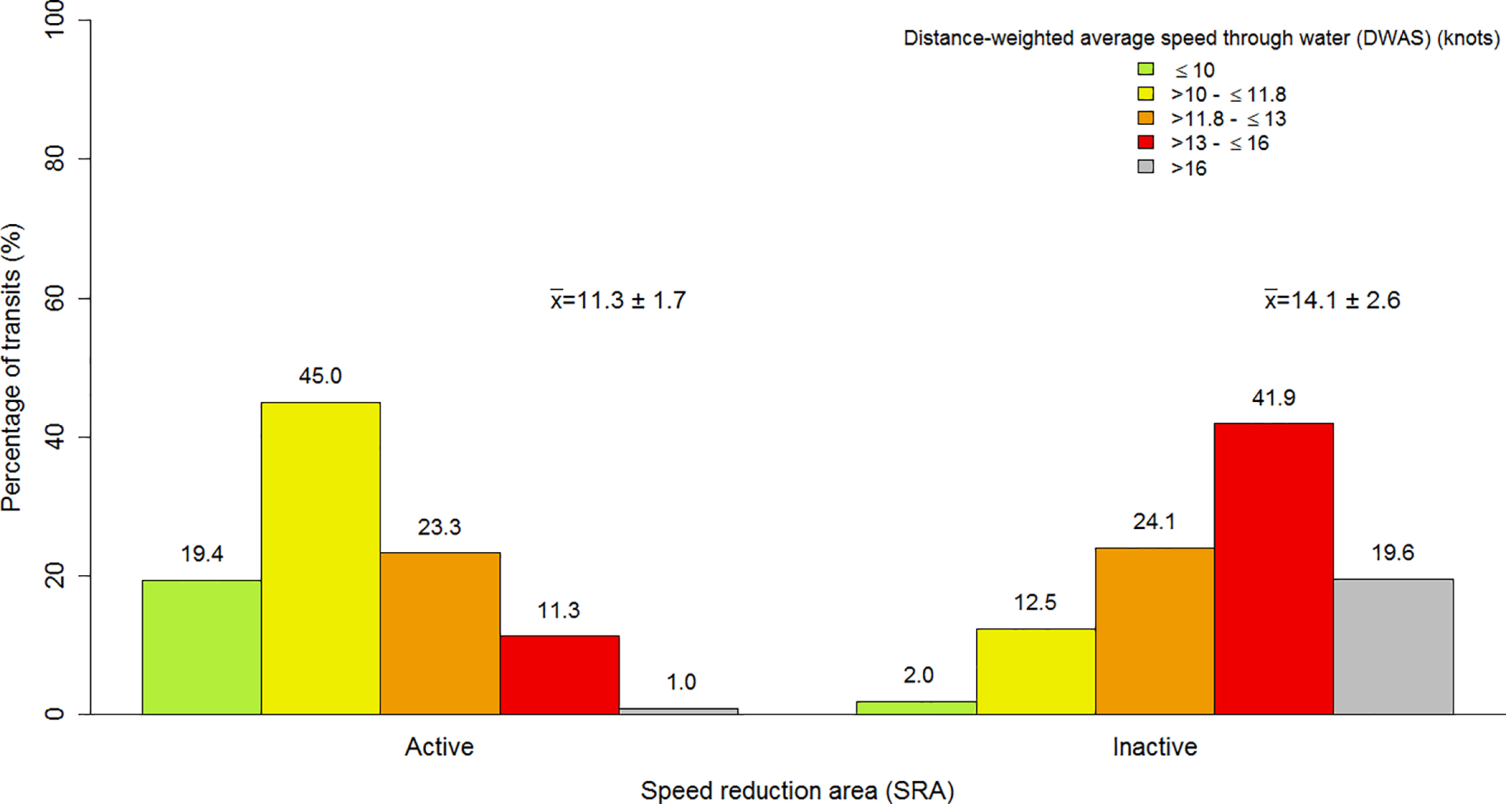
**Histograms of transits distance-weighted average speed in the speed reduction area when the voluntary measures were active (left panel) and inactive (right panel) for the 2012–2016 period.** The difference between these distributions is statistically significant (Kolmogorov-Smirnov test, D = 0.538, p-value < 0.001). The 11.8 knot threshold corresponds to a 50% probability of lethal injury in case of a collision [16].

Overall, the implementation of the SRA with the recommendation to navigate at a STW of 10 knots or less had a significant effect (p-value < 0.001) on average ship speed (Table 1). When the voluntary measures were inactive, average ship speed (DWAS) was 14.1 (14.0–14.1) knots, decreasing by 2.8 (2.7–2.9) knots when the voluntary measures were active (Table 1, Fig 3).

While the 10-knot speed limit was recommended in the SRA, 64.4% of all transits had a DWAS less than 11.8 knots compared to 14.5% when the measures were inactive (Fig 3). We also observe that the percentage of fast transits with DWAS greater than 16 knots in the SRA decreased from 19.6% when the measures were inactive to 1.0% when the speed limit was active in the SRA (Fig 3).

#### Inter-annual variation of transits speed and continuous improvement

In 2013, when SRA was implemented, transits DWAS dropped significantly (p-value < 0.001) by 2.4 knots compared to 14.0 knots when measures are not active (Table 2). Subsequently, from 2014 to 2016 when measures were active, DWAS decreased every year by 2.7, 2.7, and 2.9 knots respectively (Table 2), suggesting an overall improvement in compliance in the SRA over the years. However, this observed annual improvement only appeared to be statistically significant in 2014 compared to 2013 (Table 3).

**Table 2:** Results of a Generalized linear mixed model highlighting the statistically significant impact of the voluntary measures on transits distance-weighted average speed (DWAS) in the speed reduction area (SRA) each year of their implementation. The reference (intercept) contains DWAS for each year when the measures were inactive (2012–2016).

	DWAS (I_5_)		
* *	*Estimate*	*Conf*. *int*.	*p-value*
**Fixed Parts**			
(Intercept)	14.0	13.8 – 14.2	< .001
2013—SRA active	-2.4	-2.8 – -2.1	< .001
2014—SRA active	-2.7	-2.9 – -2.4	< .001
2015—SRA active	-2.7	-2.9 – -2.6	< .001
2016—SRA active	-2.9	-3.0 – -2.7	< .001

**Table 3:** Results of the non-parametric Kolmogorov-Smirnov (KS) test to verify if the compliance improvement (distance-weighted average speed-DWAS) in the speed reduction area is statistically significant from year to year. The “>” sign indicates that we tested if transits DWAS of the left-side year are greater than the right-side year.

Year comparison	D value (metrics of the KS test)	p-value (KS test)
2012 > 2013*	0.518	< 0.001
2013 > 2014*	0.085	0.014
2014 > 2015	0.012	0.738
2015 > 2016	0.016	0.530

* indicates statistical significance (α = 0.05) based on p-value.

**Fig 4:**
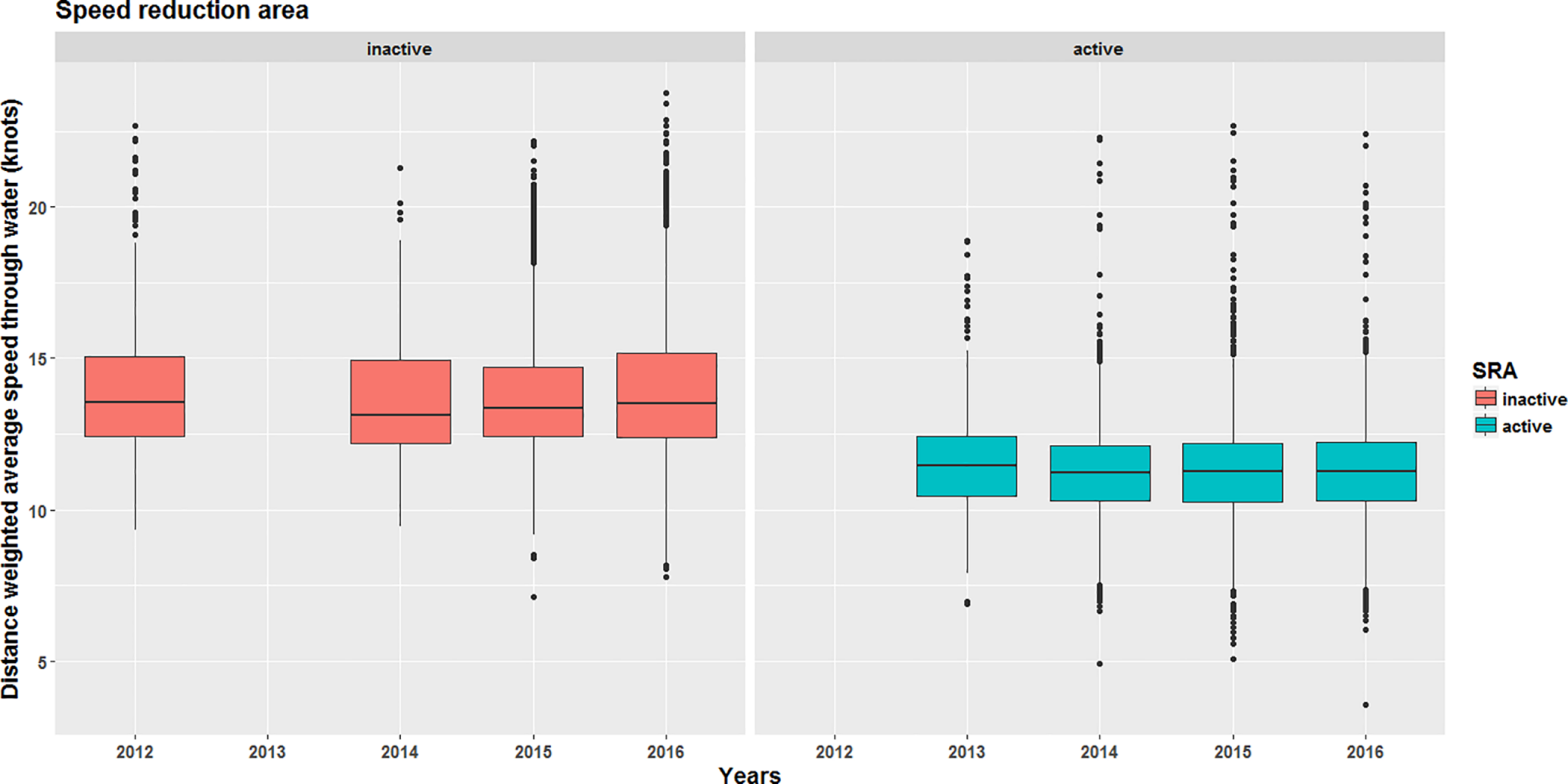
**Boxplots of ship transits distance-weighted average speed in the speed reduction area from 2012 to 2016 when the voluntary measures were inactive (left panel) and active (right panel).** The number of yearly ship transits considered are reported in Table 4.

Speed reductions were statistically significant (p < 0.001) for each year from 2013 to 2016 when the SRA was active compared to the periods when they were not (Table 2). A reduction in the length of upper whiskers (excluding outliers represented by dots) since 2013 (Fig 4) also highlights the decrease in the number of fast transits in the SRA, although transits above 15 knots are still observed (*cf*. dots on top of upper whiskers).

#### Overall compliance and efforts

We found that between 9.3% and 11.2% of transits in the SRA strictly complied with the 10-knot STW recommendation (indicator I_1_) when measures were active (Table 4). In comparison, between 0.9% and 5.5% of transits maintain their STW at 10 knots or less when the SRA was inactive (Table 4).

**Table 4:** Description of the data and indicators of overall compliance with the 10-knot speed through water limit recommended in the speed reduction area (SRA).

Year	Active measures	Measures status	Transits (number)	Vessels (number)	*I*_*1*_ (%)	*I*_*6*_ (%)	*I*_*7*_ (STW, knot)
2012*	none	na (before measure implementation)	346	187	1.2	95.3	14.3
2013*	SRA, NGA	Active (June to October)	356	187	9.3	63.7	12.4
		Inactive	nd	nd	nd	nd	nd
2014	SRA, NGA, RR	Active (May to October)	1635	522	11.2	62.0	12.2
		Inactive	109	94	5.5	90.2	14.1
2015	SRA, NGA, RR	Active (May to October)	2859	698	9.7	61.6	12.2
		Inactive	1160	444	0.9	95.8	14.2
2016	SRA, NGA, RR	Active (May to October)	2406	639	10.7	62.8	12.2
		Inactive	1958	562	1.8	94.7	14.4

* Data are only available for the month of August. The entry ‘na’ stands for “not applicable”, and ‘nd’ for “no data”. All indicators are described in the Methods section.

From 2013 to 2016, during the period when voluntary measures were active, between 61.6% and 63.7% of the total distance travelled by all transits in the SRA (I_6_ in Table 4) were covered while cruising at average speeds between 12.2 and 12.4 knots (I_7_ in Table 4). This indicates that even when compliance was not met in the SRA, ships tended to notably reduce their speed compared to when the measures were inactive (from 14.1 to 14.4 knots, I_7_ in Table 4).

Additional analyses to characterize the slow-down effort while entering the SRA (see S3 File) indicate that 64.4% of ship transits slowed down by one knot or more when entering the SRA, confirming a positive response from most mariners and pilots.

#### Factors influencing compliance: The pilotage effect

Table 5 presents the results from a GLMM aimed at identifying the factors that influence the level of compliance (i.e. ship DWAS) in the SRA when the voluntary measures are active. The results indicate that two of the tested factors have a statistically significant impact on ship speed (p-value < 0.001). First, the presence of a pilot from the CLSLP onboard during a transit has a significant impact on ship speed, with a DWAS of 0.8 knots slower than for ships without CLSLP pilots (Table 5). Second, transits going downstream are slower (through water) by 0.8 knots than those going upstream (Table 5). Finally, passenger ships appear to be significantly faster than tankers (included in the intercept of the GLMM) by 0.2 knots (p-value = 0.002). Ship’s flag (~ country of registration) does not have any significant impact on transit DWAS in the SRA (p-value = 0.113). Overall, expert pilots from the CLSLP who are assigned to more than 90% of the transits appear to be key in the achievement of speed compliance in the SRA (Table 5).

**Table 5:** Results of a Generalized linear mixed model highlighting the effects of external factors on transits distance-weighted average speed (DWAS) in the speed reduction area when measures are active.

	DWAS (I_5_) when measures are active		
* *	*Estimate*	*Conf*. *int*.	*p-value*
**Fixed Parts**			
(Intercept)	12.5	12.1–12.9	< .001
Pilot onboard (yes)	-0.8	-1.2 –-0.4	< .001
Ship’s flag (international)	-0.1	-0.2–0.0	.113
Ship class (cargo)	0.1	0.0–0.2	.096
Ship class (passenger)	0.2	0.1–0.4	.002
Transit direction (downstream)	-0.8	-0.8 –-0.7	< .001

### No-go area (NGA)

**Fig 5:**
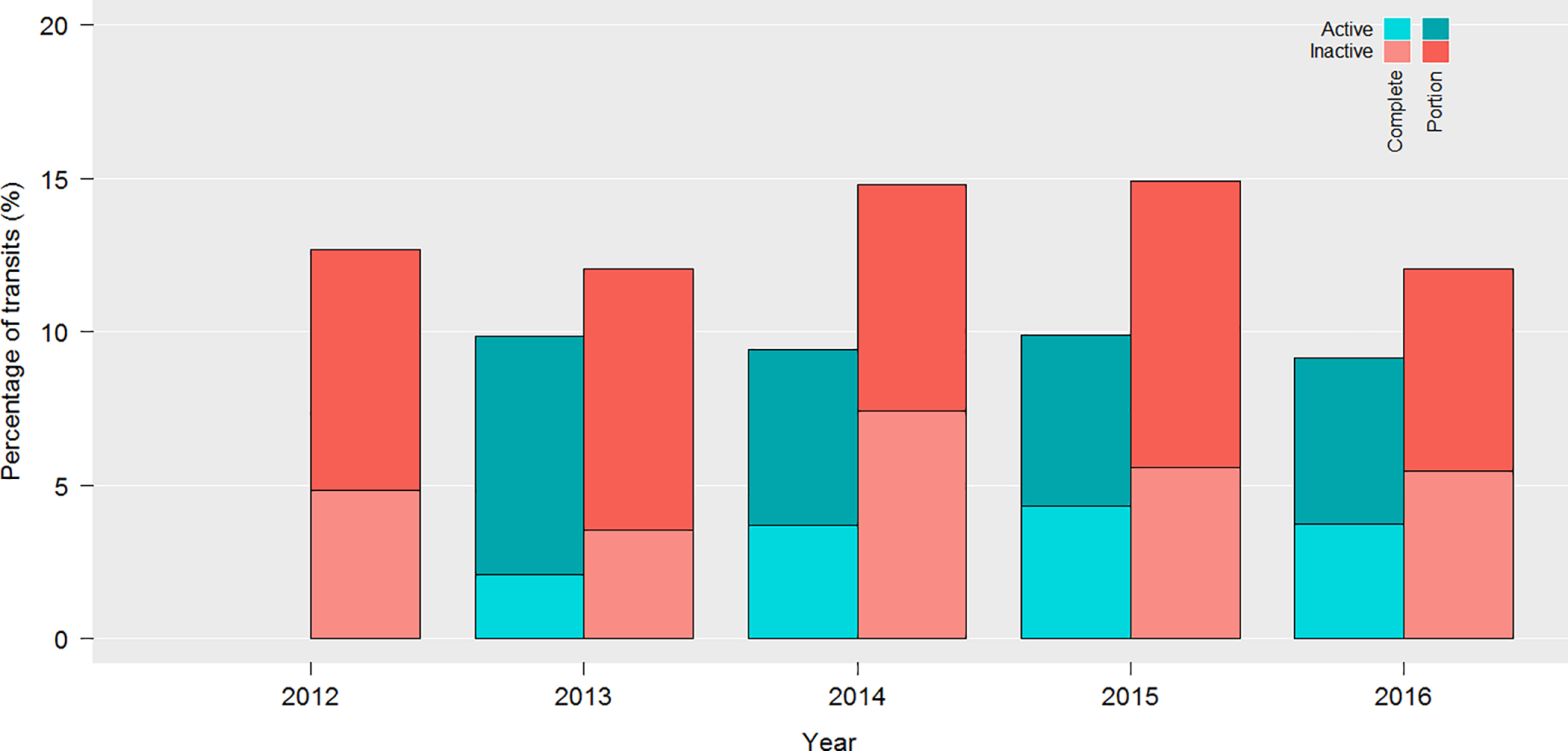
Proportion of ship transits using the no-go area completely (dark) or partially (light) during the active (blue) and inactive (red) period of the measures from 2012 to 2016.

The proportion of ship transits using at least partially the NGA during the active period was slightly lower (9.2% to 9.9%) than when the recommendation of area avoidance was inactive (12.0% to 14.9%) (Fig 5). The proportion of transits using completely the NGA was also slightly lower (2.1% to 4.3%) during the active period than when the area avoidance recommendation was inactive (3.5% to 7.4%).

**Fig 6:**
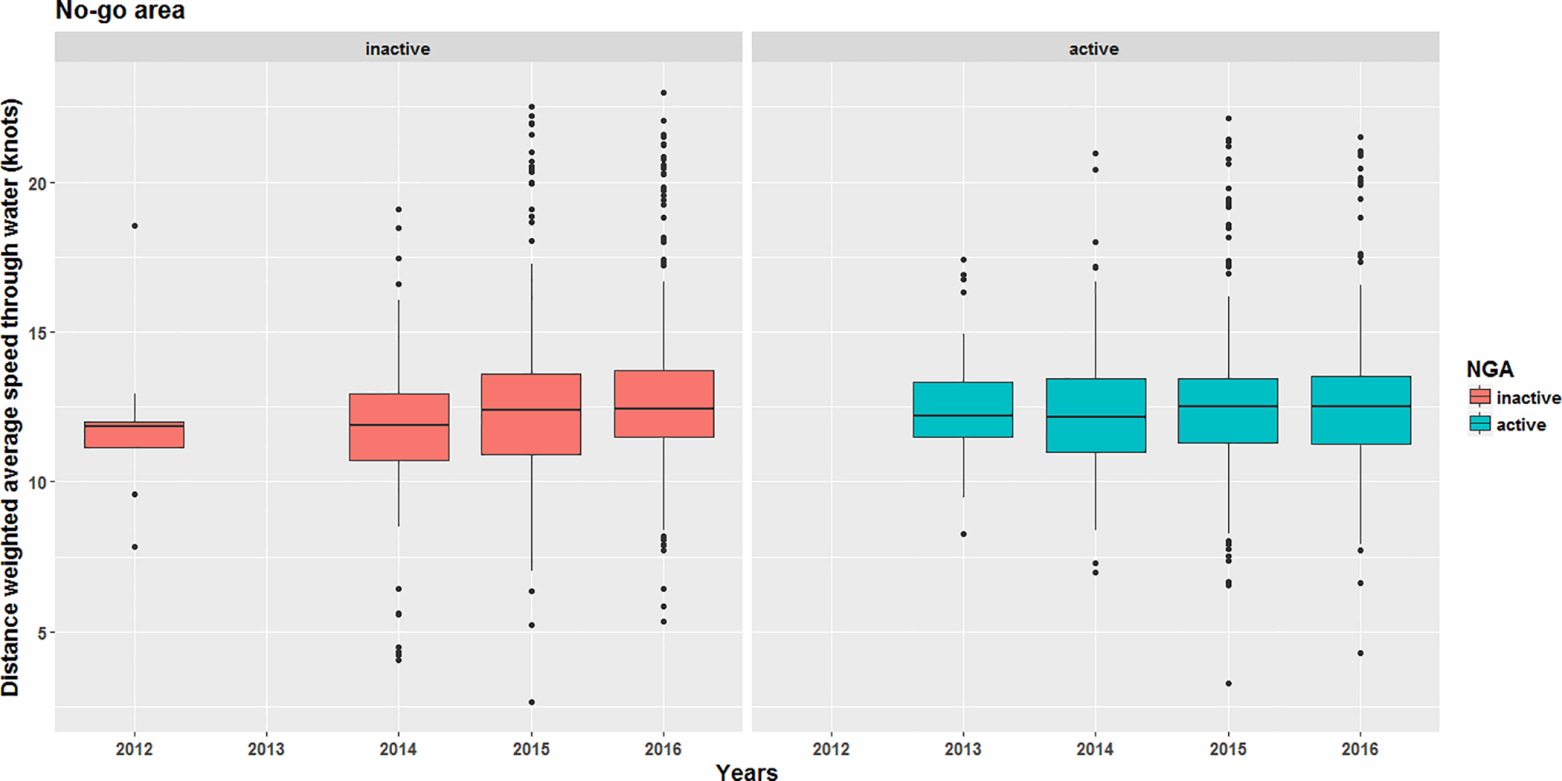
**Boxplots of transits distance-weighted average speed within the no-go area during the 2012–2016’s inactive period (left panel) and the 2013–2016’s active period (right panel) of the voluntary measures**.

While the 10-knot speed limit was recommended for vessels not avoiding the area, 62.5% and 63.5% of transits were made at DWAS over 11.8 knots when the voluntary measures were active and inactive, respectively (Fig 6). The difference was not statistically significant (Kolmogorov-Smirnov test, D = 0.043, p-value = 0.399), indicating that the recommendation to slow down at a speed lower than 10 knots had no effect on mariners’ behavior in the NGA. During the active period, transits DWAS were greater in the NGA (Fig 6, right panel) than in the SRA (Fig 4, right panel), supporting the conclusion of a weak compliance with the speed limit in the NGA.

### Recommended route (RR)

**Fig 7:**
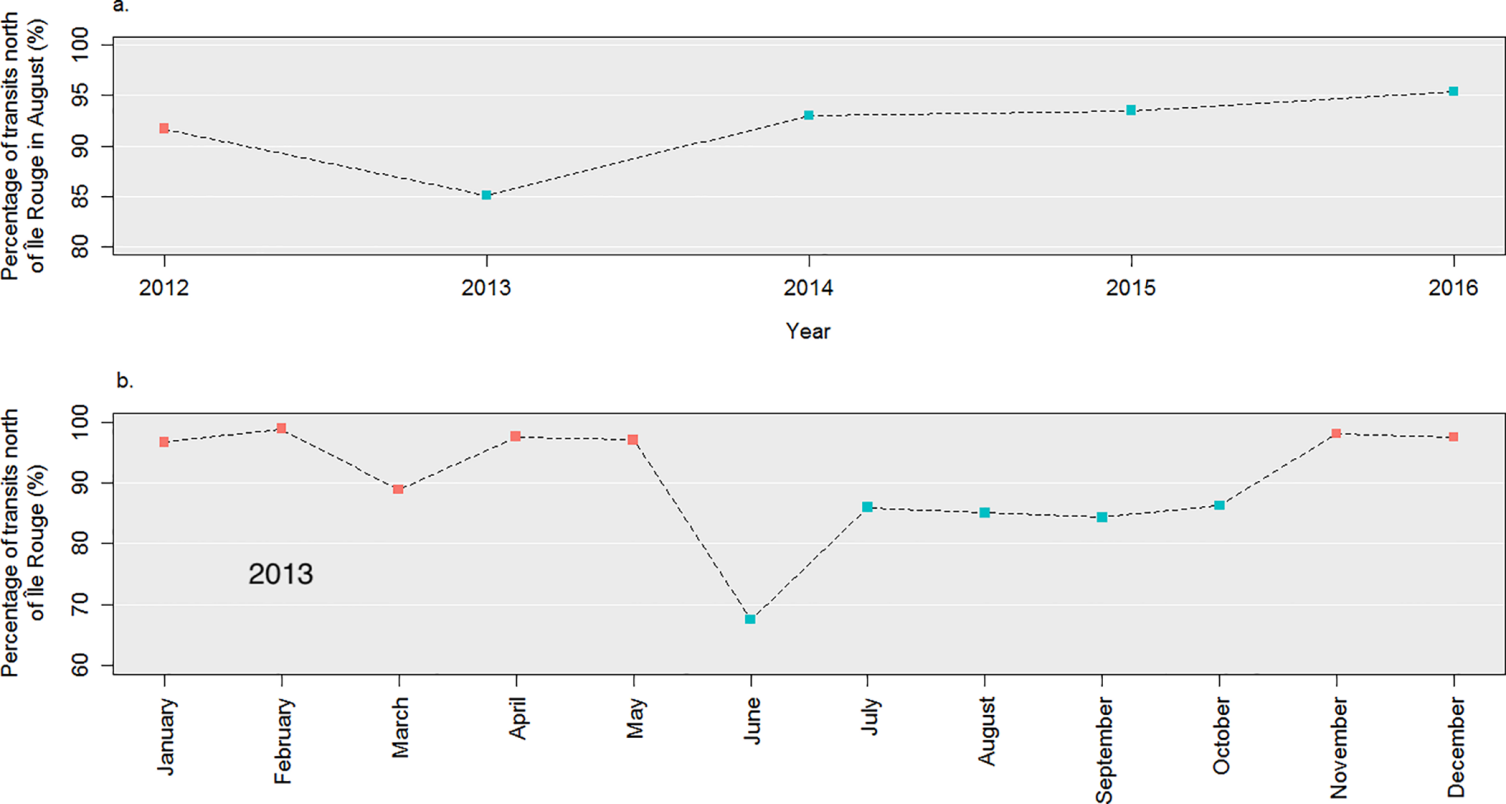
Proportion of transits complying with the recommended route (RR) (i.e. travelling north of Île Rouge). Panel a shows the percentages of transits north of Île Rouge (RR) during summers of 2012 to 2016 and panel b shows monthly percentages in 2013. Red dots indicate the periods when the voluntary measures are inactive whereas blue dots indicate periods when they are inactive.

Since 2014, the set of voluntary measures includes a recommendation to ship operators to travel north of Île Rouge (Fig 2) and stay away from sensitive areas of the beluga’s critical habitat located near the south shore [31]. Historically, the proportion of transits made along the northern route has been steadily greater than 90%, given that they result in shorter transit times and distances compared to those made using the southern route. However, to ensure safe navigation, large ships can be asked to travel south of Île Rouge where the bathymetry is deeper (*e*.*g*. Tankers with a draft greater than 15 meters), as well as when visibility is poor because it is usually less busy than the north route. The introduction of the voluntary measures to slow down vessels first made the southern route an appealing option for faster ships as it allowed to bypass the SRA. Although this side-effect was not intended in the first version of the voluntary measures released in June 2013, we observed a sudden decrease in the proportion of ships travelling north of Île Rouge from 97.7% in May 2013 to 67.5% in June 2013 (Fig 7, panel b). This unintended and unwanted response from the maritime industry was attributed to a confusion in how the recommendations were communicated. It was quickly rectified as illustrated by a subsequent increase of the proportion of transits north of Île Rouge to 86.0%, as of July 2013 (Fig 7, panel b).

After this episode, the working group requested an official Science Advice from Fisheries and Oceans Canada to determine the impact of rerouting traffic to the south shore on beluga whales and their habitat. Taking stock of relevant data available at that time, the scientists reinforced the recommendation to travel north of Île Rouge [52]. This led to the official recommendation to navigate north of Île Rouge (*cf*. RR in Fig 2) [42]. A return to normalcy was then observed as of 2014, the proportion of ships transiting north of Île Rouge even exceeding historical levels with 93.0% (2014), 93.5% (2015), and 95.4% (2016) when the RR was active (Fig 7, panel a).

### Impact assessment

#### Effectiveness assessment: Relative risk of lethal collisions

**Fig 8:**
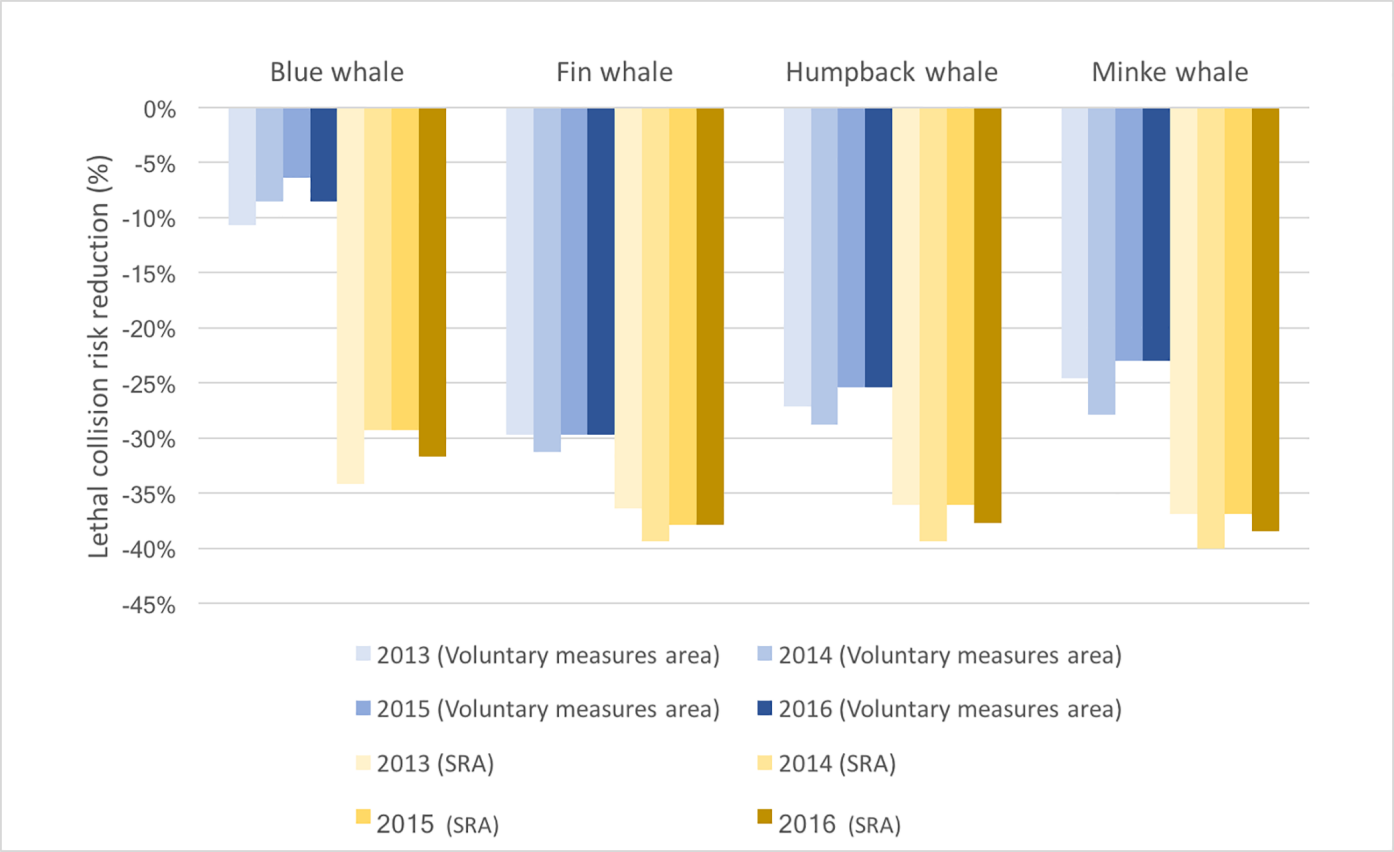
Reduction in the risk of lethal ship strikes for the four most abundant baleen whale species relative to the whole area of the voluntary measures (blue shades) and to the speed reduction area only (yellow shades) compared to August 2012 (*i*.*e*. during the “whale season” before the implementation of the voluntary measures).

We calculated the relative risk of lethal ship strike (for large vessels with AIS transponders) in the whole area covered by the voluntary measures and in the SRA only (Fig 8), which is a hotspot for the target whale species (Fig 1). The implementation of voluntary measures resulted in a decrease in risks of lethal collisions between a ship and a whale by up to 31.7% with an average of 22.5 ± 8.7% (Fig 8). The maximum gain is obained for the fin whales (risk decreases by 28.7% to 31.7%) followed by humpback whales (25.4% to 29.5%) and minke whales (23.0% to 27.2%) whereas the blue whales benefit less than the other three species (6.4% to 10.6%). In the SRA only, where co-occurrences between ships and whales are the highest (Fig 1), the risk of a lethal ship strike was reduced by up to 40.0% with an average of 36.1 ± 3.4% (Fig 8). The maximum gain is obtained for minke whales (risk decreases by 36.9% to 40.0%) followed by fin whales (36.4% to 39.4%) and humpback whales (36.1% to 39.3%) whereas the blue whales benefit less than the other three species (29.3% to 34.2%).Let’s note that assuming a compliance of 100%, the voluntary measures would lower lethal collision risks by up to 45.3% in the whole area and 57.6% in the SRA, the maximum theoretical benefit being for fin whales [61]. Overall, the species that benefited the most from compliance with the voluntary measures was the fin whale, a population considered *of special concern* under the Canadian Species at Risk Act [29]. This is the species for which most cases of collisions or fresh injuries have been reported both worldwide and in the Marine Park (Ménard, data published in [1]). Conservation benefits from the speed reductions for humpback and minke whales were similar to those described for fin whales. However, they were lower in the case of the *endangered* blue whale (Fig 8).

#### Ship transit time

The conservation gains in terms of reducing collision risks were mostly obtained through compliance with the speed limit in the SRA. This resulted in an increase in ship transit time through the whole area that we evaluated for 2016 only due to data availability. Overall, we observed an increase by about 7 minutes of the average ship transit time through the area covered by the voluntary measures in 2016 compared to August 2012 due to compliance with the voluntary measures. Although ships heading upstream take more time to cover the distance than those travelling downstream by more than 10 minutes due to surface currents, the increase in transit time due to compliance with the voluntary measures is similar for both directions [62].

## Discussion

Our study illustrates how voluntary measures can be successfully applied to mitigate ship strike risks and enhance whale conservation (Table 1 and Fig 8). Contrasting with other case studies that reported a lack of response of the maritime industry to voluntary speed limits [23,28], our results demonstrate that it is possible, under a given set of conditions, to achieve significant gains in wildlife conservation without resorting to enforced regulations, coercion or monetary incentives.

In this section, we draw lessons from this ongoing voluntary collaborative endeavor to protect whales and report on some important advantages of a voluntary framework. We further discuss some factors that arguably contributed to the overall success of the approach in an effort to inform the ongoing debate about when, where and how voluntary measures can be successful [18].

### Overall remarks on compliance

Overall, the greatest behavioral change from the maritime industry was observed in the SRA (Table 1, Fig 3 and Fig 4), with a weaker compliance in the NGA (Fig 5 and Fig 6). The absence of relationships between changes in behavior in the SRA and ship’s country of registration or ship class (Table 5), along with the weaker compliance observed in the NGA located outside of the mandatory pilotage area (*cf*. downstream of the pilot station in Fig 2) confirm that the presence of pilots from the CLSLP onboard transiting ships was the main factor driving compliance with speed limit reductions in the SRA (see GLMM results in Table 5). Pilotage is mandatory for all ships affected by the measures. However, Canadian shipping companies that regularly transit through the region may encourage their captain to obtain a pilotage certificate for their vessels, and not be required to have on board a pilot from the CLSLP. The presence of a representative of the CLSLP within the working group insured that recommendations about voluntary measures were transferred to the pilots through internal communications. Chances of compliance were further enhanced by the integration of the voluntary measures to the charts displayed on navigation systems of the pilots (*Pilot portable unit*).

Another factor that played a significant role in the compliance in the SRA is the direction of transit. Interestingly, the transits heading upstream were significantly faster (through water) than those heading downstream (Table 5). This is explained by the location of the pilot station compared to the SRA (Fig 2). In fact, when a pilot gets onboard a ship going upstream (to the south) at the pilot station, it takes him several minutes to exchange all useful safety information with the ship’s captain and crew to be able to ensure safe navigation during the whole transit. By the time all safety information have been exchanged, a ship may have entered the SRA without slowing down, explaining the greater DWAS observed for upstream transits (Table 5).

Factors that might explain the lower responsiveness of vessels in the NGA outside both the mandatory pilotage area and the marine park include a lower level of awareness, a greater cumulative impact of the voluntary measures on transit time for Canadian companies which frequently use of this area, seamanship, or a reluctance to change a well-established routine. Further analyses showed that close to 100% of the transits not avoiding the NGA as recommended were from Canadian flagged ships, the clear majority of which being owned by only three different companies (Pers Comm).

### Voluntary agreement: Flexibility, adaptiveness and responsiveness

An advantage of bottom-up voluntary agreements over top-down regulatory processes is their flexibility and the relative ease with which they can be revised and updated as new knowledge becomes available [20,21]. This is illustrated in our case study by the quick response observed from mariners at the end of June 2013, when they were instructed to navigate north of Île Rouge to avoid circulating within sensitive areas of the endangered St. Lawrence beluga population’s critical habitat (Fig 7, Panel b). The flexibility of the voluntary framework allowed to add this recommended route RR as a new measure at the beginning of the next season without going through a heavy revision process Fig 2 [63]. In the same vein, minor modifications were also made to both the SRA and the NGA, agreed upon by members of the working group. Such adjustments would have been hardly achievable in a timely fashion through a formal regulatory process which would have likely been more complex and too time-consuming.

Another quick adjustment was made to the limits of the SRA and NGA off the pilot station (Fig 1). Prior to the 2016’s active period, the working group proposed to modify the upstream limit of the NGA as it was overlapping with a portion of the precautionary area nearby the pilot boarding area. This change was made in a single meeting and printed on maps for the 2016 season.

### Factors of success

According to informal feedbacks from the working group members, several factors contributed to the successful adoption of the voluntary measures. They are presented below in no specific order.

#### Direct communication

The presence of a mandatory pilotage area was a major advantage in enhancing compliance (Table 5). By having a representative of the pilotage corporation as a regular member, the working group could disseminate a conservation message to a cohort of around 80 local pilots very effectively. Without such an organization, the working group would probably have had to deal with numerous international associations or companies separately. The presence of the pilots allowed avoiding the scale mismatch issue often observed in the management of social-ecological systems [64] (*e*.*g*. an issue encountered while attempting to mitigate collisions off the coast of southern California [55]). Second, as many pilots come from local communities, their awareness of conservation issues with these local whale populations was likely higher, and may have played a role in the early-adoption and sustained compliance with the voluntary conservation measures. Third, reducing collision risks with whales is in line with the mandate of the pilotage corporation to ensure safe navigation in the SLE. Damages to vessels and crew injuries may occur as a result of a ship strike [1,65]. Moreover, the reduction of ship speed in areas where whales and whale-watching vessels aggregate [34] also has the benefit of reducing collision risks between ships and smaller crafts. This might also contribute as a natural incentive for compliance in this busy estuarine social-ecological system.

#### Historical context of the agreement

Some members of the working group were part of an already existing concertation table called the *Comité Concertation Navigation* dedicated to sustainable navigation, providing a fertile ground for a subsequent collaboration on the collision risks issue. Literature and previous case studies on sustainable collaborative management have emphasized the importance of communication and trust between stakeholders [66]. The prior existence of these relationships and the small number of stakeholders likely contributed to the successful implementation of these voluntary measures.

The existence for almost 20 years of the Saguenay–St. Lawrence Marine Park encompassing the proposed voluntary SRA, and the presence of representatives of this federal agency on the working group have also contributed to the quick adoption of the measures by the maritime industry. However, the overlap of the marine park with the mandatory pilotage area makes it difficult to disentangle the relative contributions of mandatory pilotage and the marine park in explaining compliance.

Finally, the timely availability of decision-support tools and models [37] to test mitigation scenarios co-constructed by the working group and predict the likely impacts, contributed to maintain a momentum and promoted scientifically informed recommendations [41,52].

#### Participatory process

From the very beginning, the functioning of the working group has relied on a voluntary commitment from all stakeholders including the private sector. Holding an average of two meetings per year allowed maintaining a regular flow of information and feedbacks between stakeholders while building working relationships. A regular member that would miss two consecutive meetings could be excluded or downgraded to an observer role, ensuring a high participation rate at each meeting. The right balance between representativeness of industry and conservation interests, as well as a manageable size for the working group was also crucial. With only five regular members representing commercial shipping on the working group (S1 Table), the voluntary conservation measures reach 100% of the ship transits in the area, particularly due to the presence of the president of the CLSLP who provides access to all pilots in the area.

The agreed upon decision-making process gives priority to consensus, which is in line with the co-construction approach adopted by the group to propose realistic solutions to mitigate collision risks [4]. Taking the time to carefully explain and discuss the collision issue within the working group without bringing any already made solutions to the table strongly enhanced members’ commitment. The co-development of solutions was thus truly a collective team effort between members of the working group searching for win-win solutions. The co-construction of realistic conservation measures allowed focusing on solutions having affordable costs for the industry, not considering the gains related to their corporate image, with clear benefits for conservation (Fig 8).

#### Management frameworks and guiding principles

The collaborative work carried out by all stakeholders to manage ship-whale interactions is part of a continuous improvement process, driven by the willingness to enhance marine mammal protection over the years. Accordingly, our results suggest a continuous improvement of compliance with the speed limit in the SRA over the years (Table 2), although the yearly improvement was not statistically significant every year (Table 3). From the very beginning of the working process, the concept of scientific uncertainty was embraced by all members, as reflected by the adoption of a risk management approach as part of an adaptive management framework [40]. Acknowledging that scientific knowledge is subject to uncertainty and evolution also supports the preference of an easy-to-update and flexible voluntary framework over a more rigid regulatory approach.

The working group also adopted sustainable development as a leading principle, recognizing the importance of promoting conservation enhancement measures that do not compromise maritime activities. Finally, concertation being at the core of the working process, all members acknowledged that the recommendations should be guided by unbiased information and sound science. This was embodied by the presence of researchers from academia and the government as regular members and resource-persons of the working group respectively (S1 Table).

#### Government commitment

The working group is co-chaired by two departments of the Federal government, namely Parks Canada and Fisheries and Oceans Canada, ensuring the provision of resources to organize meetings, to access and collect data, and to monitor compliance. The latter required the purchase of equipment and payment for consulting services to assess compliance and effectiveness of the voluntary measures, and address emerging issues during the work process. This was key since compliance and effectiveness monitoring are critical steps to maintain community commitment that have been pointed out as common weaknesses of voluntary agreements [18,67]. Finally, the leading position of the government certainly contributed to give credit to the process in everyone’s eyes and to maintain active participation from all members.

### Challenges and avenues for improvement

The collaborative work carried out by all stakeholders to manage ship-whale interactions is part of a continuous improvement process. Consequently, despite the encouraging results obtained so far (Table 1), the compliance level has reached a plateau in the SRA (Table 2 and Table 3) and is still low in the NGA (Fig 5 and Fig 6). Overall, we reached similar compliance results in the SRA than those reported for US eastern mandatory seasonal management areas where the same 10-knot limit applies [23], illustrating the success of our voluntary framework and participatory approach. However, the weak compliance observed in the NGA appears unsatisfactory when compared for instance to the 71% achieved in the Roseway Basin’s area to be avoided [10], highlighting the need for additional efforts to promote these measures outside the mandatory pilotage area. These observations highlight that there is room for improvement, calling for follow-up actions to increase adherence from the maritime transportation sector.

Many of the maritime transportation companies affected by the voluntary measures adhere to voluntary environmental certification programs, mainly the one promoted by Green Marine, a North-American non-profit organization and member of the working group (S1 Table). Maritime companies adhering to the Green Marine program are required to adopt environmental behaviors that go beyond what is required by regulations regarding several environmental issues. Therefore, companies’ compliance with voluntary conservation agreements such as the conservation measures proposed by the working group is consistent with such certification programs, thus improving the corporate image and competitiveness. The companies that adhere to such programs must also demonstrate that they constantly improve their environmental performance over time to keep their certification. Green Marine started to reward companies for their compliance with whale protection measures in 2017 but their earlier presence as a regular member of the working group has sent a clear message that whale protection matters. In 2017, their revised certification program integrated an indicator that rewards companies for elaborating a marine mammal management plan focusing on the reduction of their impact in sensitive areas. This environmental certification program can thus be a promising tool to further enhance compliance with the voluntary measures.

Outreach is of paramount importance in the context of voluntary agreements [68]. Future efforts to raise awareness should target companies and individual pilots that regularly disregard the voluntary measures and those showing reluctance to change, which would also contribute to prevent the “free-riding effect”. Knowing the motivations of outlier companies (*i*.*e*. fully compliant companies and those not compliant at all) could also reveal underlying mechanisms of compliance and inform further reinforcement actions.

The voluntary measures still have a provisional status. Therefore, they are not broadcast in real-time on onboard navigation systems (although they are on pilots’ portable unit). As a result, captains cannot accurately visualize the spatial limits of the SRA or NGA in real-time. This communication deficiency is a source of inaccuracy that prevents mariners from anticipating the exact location where they should start and stop complying with the voluntary measures. Another issue related to real-time information access for mariners is the lack of availability of their STW on their navigation systems (note: ship STW is not currently provided by the AIS signal). Without a real-time access to this information by both mariners and local authorities, it is not fair to use strict compliance as an indicator to characterize mariners’ behaviors in the SRA or the NGA. Moreover, broadcasting the voluntary measures via the electronic nautical charts would be key to ensure that captains can take them into account when making navigation decisions. To ensure that the broadcasting of real-time information reaches mariners at sea, it is critical to use communication tools already part of their toolbox [69].

Regarding the evaluation of the measures’ effectiveness, whereas shipping data are available through AIS on a yearly basis in our study area, we rely on an historical 25-year dataset for whale distributions. Although the use of this whale dataset is consistent with the materials used all along the multi-stakeholder participatory process [4], a yearly updated portrait of whale species distribution and abundance along with dynamic real-time whale movement would improve the accuracy of effectiveness assessment.

Finally, the implementation of statutory rules is usually made in conjunction with the provision of funds and resources for law enforcement and effectiveness assessment. Given that voluntary agreements are not based on a regulatory framework, access to resources (*e*.*g*. funds, equipment, expertise) in the long-term might be challenging, affecting the capacity to promote compliance and monitor effectiveness. The Government’s commitment to ensure the provision of resources in the context of voluntary agreements is therefore key to promote the success and widespread use of this alternative conservation avenues.

## Conclusion

The traditional regulatory approach to conservation is often blamed for its focus on deterring negative behaviors, doing nothing to encourage and reward positive ones [19]. We presented a case study where a voluntary agreement between the public and private sectors, NGOs, and academia led to a significant reduction in collision risks and thus, improvement of wildlife conservation for various whale species including species at risk. In agreement with the broader literature on voluntary agreement for conservation, the benefits of the voluntary measures implemented in the St. Lawrence Estuary include the pro-active commitment from the maritime industry (which is likely to reduce conflicts with regulators), the greater flexibility and freedom that allowed to come up with cost-effective and tailored-made mitigation measures, and the fast achievement of conservation gains [20,21]. In the context of ship-whale collision risk mitigation, our results challenge the conclusions of previous experiments in other regions about ineffectiveness of voluntary agreements [28]. This emphasizes the importance to continue feeding the debate about where, when and how voluntary agreements can be effective frameworks for conservation [18].

In the present case study, some key factors contributing to the success have been pointed out. The pre-existence of decision support tools, institutions, and relevant scientific knowledge allowed to maintain the momentum of the work process. The co-construction approach embodied in the building of conservation measures by all stakeholders allowed realistic conservation measures to be proposed, while accounting for operational constraints of the maritime industry. According to economic theories, the motivation of the private sector to bear the costs from adopting conservation measures not associated with monetary incentives needs to come from other compensatory benefits [70]. In our study, the costs of complying with the voluntary measures remained low on average for the industry (*i*.*e*. small increase in the duration of transits). However, the slow-down effort was rewarded by some co-benefits and positive externalities such as the benefits for the corporate image of the maritime transportation industry. Other positive externalities of slowing down in the SRA include the improvement of safety at sea by reducing the collision risks between ships and whale-watching vessels.

Acknowledging that theories of economic rationality have been shown to be unable to explain some decisions made by private organizations, we put forward the importance of working with stakeholders willing to promote environmentally-friendly behaviors beyond their own mandate. Statements from local representatives of the private sector putting forward their personal environmental concerns have been frequent during the working process. Although the sense of place has been identified as a psychological factor contributing to compliance, the individual willingness to make a difference for the environment in times when the human footprint on Earth’s ecosystems has already gone beyond some reversible limits might also explain the motivation of corporate representatives to champion conservation endeavors.

Beyond the multiple factors that can contribute to explain the success of this voluntary conservation approach, confidence and trust built between all stakeholders were certainly stepping stones achieved through free and open communication and transparency. This successful collaborative experience provides a human and working capital on which to build upon to solve other issues such as the chronically high levels of anthropogenic underwater noise in whale habitat along with potential shifts in the marine ecosystem as a result of climate change.

## Supporting information

S1 Table Composition of the working group. (DOCX)

S1 File Speed through water: Description and accuracy. (DOCX)

S2 File Step-by-step procedure to compute the effectiveness of the voluntary conservation measures. (DOCX)

S3 File Overall slow-down efforts in the speed reduction area (SRA). (DOCX)
