# Supplementary material for: A voluntary conservation agreement reduces the risks of lethal collisions between ships and whales in the St. Lawrence Estuary (Québec, Canada): From co-construction to monitoring compliance and assessing effectiveness
Source: PLoS One. 2018 Sep 21;13(9):e0202560. doi: 10.1371/journal.pone.0202560 (PMC6150506; doi:10.1371/journal.pone.0202560)
Supplement: S1 Table — (DOCX) [file pone.0202560.s001.docx]

## S1 Table. Composition of the working group

| **Sector** | **Institution** | **Status (membership)** |
| --- | --- | --- |
| Commercial shipping | Corporation of the Lower St. Lawrence Pilots | Regular member |
|  | Green Marine | Regular member |
|  | Shipping Federation of Canada | Regular member |
|  | St. Lawrence Economic Development Council | Regular member |
|  | St. Lawrence Shipoperators | Regular member |
| Expert organizations on Marine Mammals | Group for Research and Education on Marine Mammals (GREMM) | Regular member |
|  | Réseau d’Observation de Mammifères Marins (ROMM) | Regular member |
| Academia | Université du Québec en Outaouais (UQO) | Regular member |
| Government | Fisheries and Oceans Canada – *Ocean Management* | Co-chair |
|  | Parks Canada | Co-chair |
|  | Fisheries and Oceans Canada – *Ocean Management* | Resource-person |
|  | Parks Canada | Resource-person |
|  | Canadian Coast Guard | Resource-person |
|  | Fisheries and Oceans Canada – *Science* | Resource-person (x2) |
|  | Transport Canada | Resource-person |
|  | Ministère des Forêts, de la Faune et des Parcs du Québec | Resource-person |
