# Supplementary material for: A voluntary conservation agreement reduces the risks of lethal collisions between ships and whales in the St. Lawrence Estuary (Québec, Canada): From co-construction to monitoring compliance and assessing effectiveness
Source: PLoS One. 2018 Sep 21;13(9):e0202560. doi: 10.1371/journal.pone.0202560 (PMC6150506; doi:10.1371/journal.pone.0202560)
Supplement: S1 File — (DOCX) [file pone.0202560.s002.docx]

## S1 File. Speed through water: description and accuracy

Given the overlap of shipping routes and whale distributions in the region, the SRA is expected to have the greatest impact on ship strike mitigation. While the monitoring of ship speed over ground (SOG) is straightforward using such tools as AIS or radar [52], SOG is influenced by the currents which are locally strong with rapid changes in space and time across the region [54] mainly due to tidal effects. Therefore, for a ship operator to maintain a constant SOG through the region, he would have to constantly adjust ship engine regime to compensate for surface currents variations, which would be contrary to the practices of good seamanship (Simon Mercier, pers. comm.). Accordingly, the working group agreed to define the speed limit to 10 knots through water (STW) in the SRA [38].

Given that no navigation or monitoring tool currently provide ship STW in real-time, the decision to fix the 10-knot limit as a STW for good seamanship reasons poses two main challenges:

- Estimation of STW by operators: Ship operators must accurately estimate their STW to comply with the 10 knots speed limit. Given that no navigation tools provide this information in real-time, estimation by mariners is subject to unmeasurable errors.
- Monitoring of STW: the absence of a system measuring ship actual STW in real-time poses the problem of compliance assessment.

To address this problem of STW estimation from ship SOG, two actions were taken: 1) a speed conversion module using prevision data from a model of surface currents was developed and integrated to the monitoring system, and 2) the match between ship STW estimated by pilots compared to the monitoring system (AIS-STW) was assessed thanks to a one-year voluntary experiment with pilots (Fig 1 and Fig 2). Accounting for AIS data precision and current model uncertainty, we came up with an uncertainty of 0.7 knots on the estimation of ship STW.


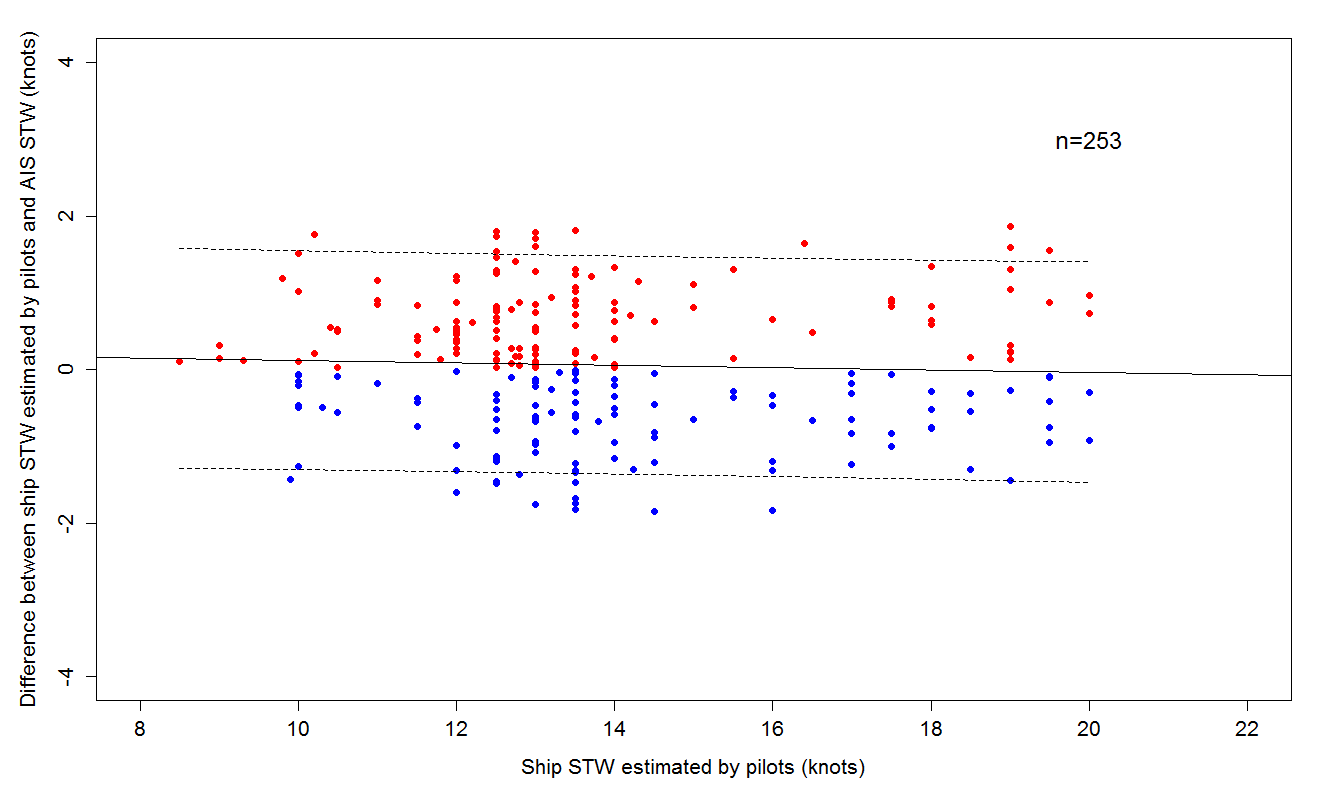


Fig 1. Difference between ship STW estimated by pilots and derived from AIS data as a function of ship speed estimated by pilots. The plot indicates that pilots do not display any systematic bias when estimating STW at low vs. high speeds.


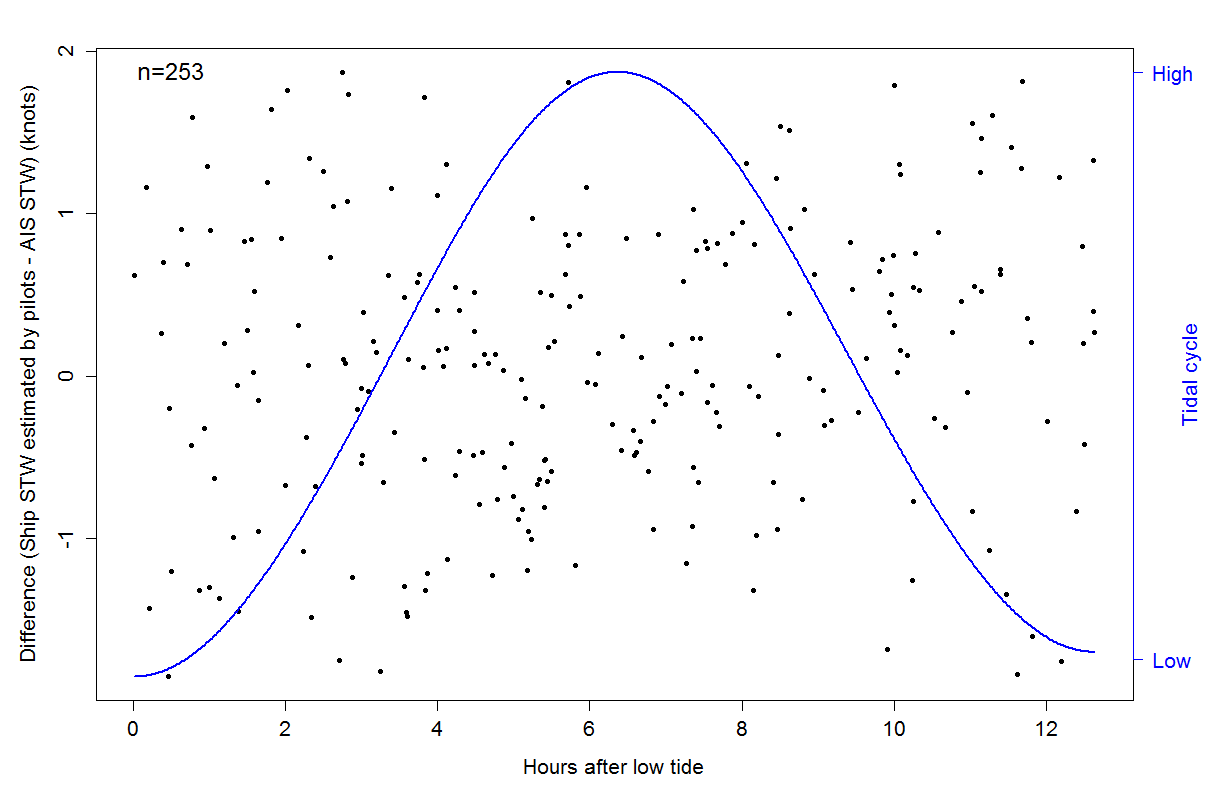


Fig 2. Difference between ship STW estimated by pilots and derived from AIS corrected with a current model as a function of tidal cycle. The plot indicates that pilots do not display any systematic bias when estimating STW according to the tidal cycle.
