# Supplementary material for: A voluntary conservation agreement reduces the risks of lethal collisions between ships and whales in the St. Lawrence Estuary (Québec, Canada): From co-construction to monitoring compliance and assessing effectiveness
Source: PLoS One. 2018 Sep 21;13(9):e0202560. doi: 10.1371/journal.pone.0202560 (PMC6150506; doi:10.1371/journal.pone.0202560)
Supplement: S2 File — (DOCX) [file pone.0202560.s003.docx]

## S2 File. Step-by-step procedure to compute the effectiveness of the voluntary conservation measures.

The following 6-step spatial analysis is performed to assess the effectiveness of operational measures for a given species:

- Build a regular grid *G* that overlays the whole study area.
- Compute the relative probability of presence of an individual from the species for each cell of *G* over the study area. This is the raster *Prel(species)*.
- Compute the relative probability of presence of a ship for each cell of *G* over the study area. This is the raster *Prel(ship)*.
- Compute the relative probability of a ship-whale co-occurrence for each cell of *G* over the study area by multiplying both rasters *Prel(species)* and *Prel(ship)*. This is the raster *Prel(co-occurrence)*.
- Compute the probability of whale being killed if a collision happens for each cell of *G* over the study area. This probability is calculated using ships’ average speed in each cell of the grid *G* applying the equation $P\left( lethal \right)=\frac{1}{1+{exp}^{-(-4.89+0.41\times speed)}}$ proposed by [13]. This equation has been preferred to the more recent relation proposed by [11] because whereas both are similar at high speeds, the latter suggests that lethal collision may occur even if ship speed is 0, which is not reasonable.
- Finally, the map of the relative risk of collision between a ship and an individual from the target whale species being lethal is computed by multiplying the rasters *Prel(co-occurrence)* by *P(lethal)*. This is the raster *RR (species)*.
