# Supplementary material for: A voluntary conservation agreement reduces the risks of lethal collisions between ships and whales in the St. Lawrence Estuary (Québec, Canada): From co-construction to monitoring compliance and assessing effectiveness
Source: PLoS One. 2018 Sep 21;13(9):e0202560. doi: 10.1371/journal.pone.0202560 (PMC6150506; doi:10.1371/journal.pone.0202560)
Supplement: S3 File — (DOCX) [file pone.0202560.s004.docx]

## S3 File. Overall slow-down efforts in the speed reduction area (SRA)

In the previous section, we drew a portrait of the overall compliance with the 10-knots STW limit in the SRA. To better understand the slow-down effort from the maritime industry and target further reinforcement actions, we mapped transit speed variation before (3 km buffer zone) *vs.* within the SRA against transit average STW (I_5_) during the active period with results reported in Fig 1 (left panel). This analysis reveals that during the active period for 2014-2016:

- 64.5 % of all transits through the SRA slowed down by 1 knot or more when entering it, including 50% who are not fully compliant;
- 31.0 % of the non-compliant transits did not slow down (*i.e.* speed change between +1 and -1 knots) including 2.1 % displaying accelerations > 1 knot;
- 4.3 % of the transits were already compliant and showed no speed change.


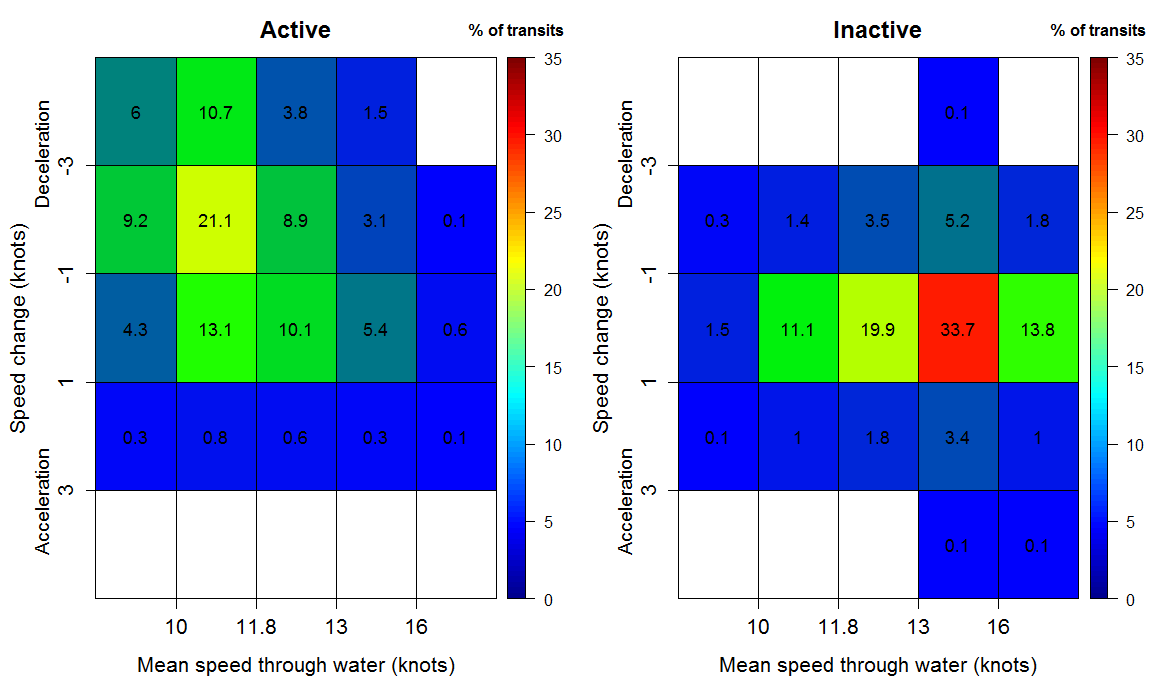


Fig 1. Speed variation 3 km before vs. within the SRA against transit’s average STW within the SRA for both the active (left panel) and inactive periods (right panel) for 2014-2016. Numbers in each cell indicate the percentage of transits.

We also note that 19.8 % of all transits travelled through the SRA at an average STW of 10 knots or less (indicator I_6_), which confirms that the strict compliance (I_1_) showing [9.7 – 11.0] % compliance is a severe indicator that does not acknowledge the efforts of the industry and the related benefits in conservation.

The slow-down efforts in the SRA noted during the active period are contrasting with what is observed when the voluntary measures are inactive (Fig 1, right panel). During the inactive period, 80.0 % of the transits do not change speed notably when travelling through the SRA with 98.1 % displaying average STW greater than 10 knots. This highlights that almost all transits are affected by the 10-knot limit recommendation when the measures are active and confirms the importance of assessing slow-down effort I_4_ and not only the strict compliance I_1_.
